# A STUDY OF CATASTROPHIC HEALTH EXPENDITURES IN INDIA - EVIDENCE FROM NATIONALLY REPRESENTATIVE SURVEY DATA: 2014-2018

**DOI:** 10.12688/f1000research.75808.1

**Published:** 2022-02-03

**Authors:** Shyamkumar Sriram, Muayad Albadrani

**Affiliations:** 1 Department of International Health, Johns Hopkins Bloomberg School of Public Health, Baltimore, MD, USA; 2 Department of Famiy and Community Medicine, Taibah University, Medina, Saudi Arabia

**Keywords:** financial protection, out-of-pocket health expenditure, catastrophic health expenditures, universal health coverage

## Abstract

Abstract

**Background:** India is taking steps to provide Universal Health Coverage (UHC). Out-of-pocket (OOP) health care payment is the most important mechanism for health care payment in India. This study aims to investigate the effect of OOP health care payments on catastrophic health expenditures (CHE).

**Methods:** Data from the National Sample Survey Organization, Social Consumption in Health 2014 and 2018 are used to investigate the effect of OOP health expenditure on household welfare in India. Three aspects of catastrophic expenditure were analyzed in this paper: (i) incidence and intensity of ‘catastrophic’ health expenditure, (ii) socioeconomic inequality in catastrophic health expenditures, and (iii) factors affecting catastrophic health expenditures.

**Results:** The odds of incidence and intensity of CHE were higher for the poorer households. Using the logistic regression model, it was observed that the odds of incidence of CHE was higher among the households with at least one child aged less than 5 years, one elderly person, one secondary educated female member, and if at least one member in the household used a private healthcare facility for treatment. The multiple regression model showed that the intensity of CHE was higher among households with members having chronic illness, and if members had higher duration of stay in the hospital. Subsidizing healthcare to the households having elderly members and children is necessary to reduce CHE.

**Conclusion:** Expanding health insurance coverage, increasing coverage limits, and inclusion of coverage for outpatient and preventive services are vital to protect households. Strengthening public primary health infrastructure and setting up a regulatory organization to establish policies and conduct regular audits to ensure that private hospitals do not increase hospitalizations and the duration of stay is necessary.

## Introduction

Goal 3 of the United Nation’s Sustainable Development agenda has the specific goal to provide universal health coverage (UHC) to its population and to improve financial protection. UHC includes securing access to quality healthcare and safe, affordable medicines and vaccines for everyone ( Saksena *et al.,* 2014). Resolution 58.33 of the World Health Assembly recommends that all WHO member states should provide UHC to their entire population and protect households from catastrophic health expenditures (CHE) ( Obermann *et al.*, 2018). Any household out-of-pocket (OOP) health spending that exceeds a certain proportion of household’s financial ability is defined as CHE ( Xu *et al.,* 2003). Globally around 100 countries have achieved UHC or initiated reforms towards UHC ( Obama, 2008; Summers, 2015). Even though most countries are striving to enable their citizens to obtain the healthcare they need without financial barriers, 808 million people still experience CHE each year ( Wagstaff *et al.,* 2018). More than 90% of the people experiencing CHE live in low-income countries ( Xu *et al.,* 2003). Financial protection needed to a population depends on their dependence on OOP health expenditure for paying for healthcare ( Xu *et al.,* 2003). Dependence of the households on OOP payments for obtaining healthcare escalates the financial burden of the households ( Amaya *et al.,* 2011; Wagstaff *et al.,* 2003; Xu *et al.,* 2003). With OOP health expenditures constituting around 62.6% of total health expenditures, India ranks third in the Southeast Asia region among countries with high OOP health expenditure ( Balarajan *et al*. 2011; Hooda, 2017; Sriram 2018).

Many studies have examined the health expenditures on specific diseases such as diabetes, tuberculosis, cancer, injuries *etc.,* but the problem was that most of these studies were done in small geographical areas of the country and their representativeness for the whole nation was limited ( Binnendijk *et al*., 2012; Yesudian *et al*., 2014; Rao *et al*., 2011; Prinja *et al*., 2015; Muniyandi *et al*., 2005; Ramachandran *et al*., 2007). Some studies have examined the determinants of OOP health expenditures for outpatient care in a few districts of India for certain age groups ( Brinda *et al*., 2012; Gupta *et al*., 2016). Also, other studies have used different National Sample Survey Organization (NSSO) datasets and other nationally available data like National Family Health Survey (NFHS), *etc.* to study disease-specific OOP health expenditures for hospitalizations ( Kastor & Mohant, 2018). Studies have looked at OOP health expenditures due to non-communicable diseases (NCDs) ( Tripathy *et al*., 2016), burden of OOP payments due to medicines ( Selvaraj *et al*., 2018), OOP health expenditure for maternal care ( Mohanty & Kastor, 2017), OOP health expenditure for accidental injury ( Pradhan *et al*., 2017), but they did not address the specific research questions related to CHE in general and factors affecting incidence and depth or gap of CHE.

The primary objective of the study is to detect the features of households, health conditions, and health delivery system issues that make people prone to CHE. In particular, the study will examine the association of households’ demographic characteristics, social structure, and healthcare utilization that appear to be associated with relatively high levels of expenditure and also quantify the burden of OOP health expenditures and CHE. In this research, we used the data from two surveys, the 2014 and 2018 NSSO data to assess the level of financial protection in India ( NSSO, 2014). To measure the effect of CHE on households, we estimate (i) incidence, intensity, and inequality in CHE in India (ii) incidence and intensity of CHE across different states in India (iii) various aspects influencing the incidence and intensity of CHE in India.

**Figure 1.**
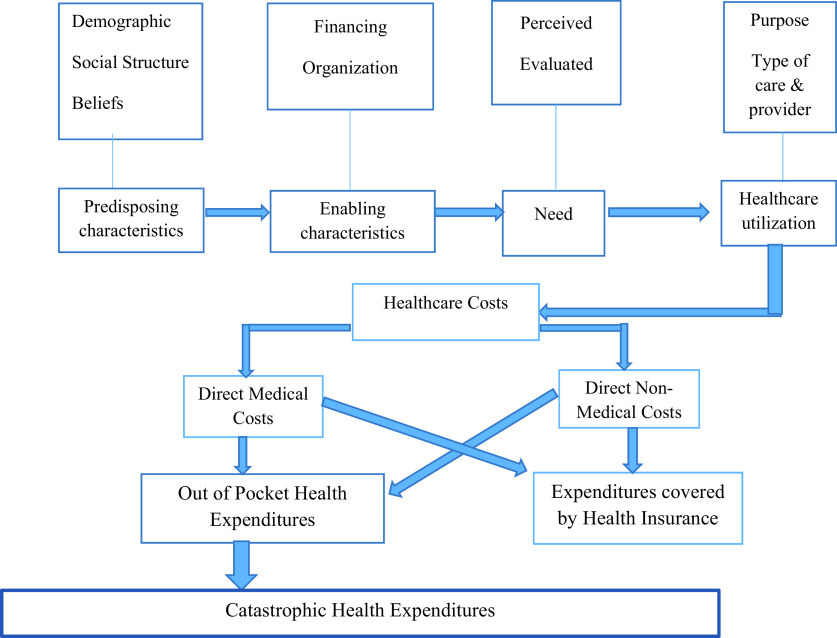
Determinants of household’s OOP health expenditures using Andersen’s behavioral model of healthcare utilization.

India is taking steps to provide UHC to its citizens. By identifying the incidence, intensity, socioeconomic inequalities in CHE, this study helps the central government provide an appropriate higher budgetary allocation for the groups that have higher OOP health expenditures and helps in designing health insurance programs that benefit the poor. This research will help in reducing catastrophic spending in India. Andersen’s Behavioral Model of Healthcare Utilization as shown in Figure 1 will be used to guide this research ( Andersen, 1995). The Andersen model examines the predisposing, enabling, need and healthcare utilization characteristics.

## Data

### Data sources

The data from the NSSO of the Government of India were used for the study. The study only used secondary data analysis obtained from the Government of India. Only deidentified data has been provided by the Government of India. No ethical approval was required since there is no human participation in the study. Social Consumption (Health), NSS 71 ^st^ Round and NSS 75 ^th^ Round were used for this analysis. Both the surveys covered whole of the Indian Union. The surveys collected data from 65,932 randomly selected households (NSS 71 ^st^ Round) and 113,823 households (NSS 75 ^th^ Round) The data for the NSS 71 ^st^ round survey were obtained from January to June 2014 and the data for the NSS 75 ^th^ round were collected between July 2017 and June 2018 ( NSSO, 2014).

## Methods

### Measuring incidence and intensity of CHE

The incidence of CHE was calculated from the proportion of OOP healthcare payments which exceed a certain threshold in relation to the household consumption expenditure ( Wagstaff *et al*., 2003). In this research, the OOP health expenditure is compared with the household consumption expenditure, and it is assumed that a household experienced CHE if health expenditure exceeds the 10% threshold level. Catastrophic payment headcount informs the proportion/number of households affected by CHE i.e., the number of households who are experiencing an OOP healthcare expenditure above 10% of household consumption expenditure.

Catastrophic payment headcount is given by the formula:

$$\mathrm{HC}=\frac{1}{N}\sum_{i=1}^{N}E$$

HC is the catastrophic payment headcount. The indicator E=1 is defined when T _i_/X _i_ >Z and zero otherwise. Here Z is 0.10. T is the household OOP health expenditure; X is the total household consumption expenditure and N is the sample size. The theoretical minimum and maximum values of catastrophic payment headcount are 0% and 100% respectively. The CHE incidence (headcount) does not indicate the degree to which the household’s CHE exceed the threshold value, thus the CHE intensity (overshoot) has also been estimated. The intensity (overshoot) of the CHE is the average degree when the household OOP health expenditures as a proportion of the household consumption expenditure exceeds the pre-specified thresholds (10%).

Average catastrophic excess (O) measures this intensity of CHE, and it is given by the formula below:

$$O=\frac{1}{N}\sum_{i=1}^{N}O$$

Oi is the excess or overshoot and it is calculated by the formula, O _i_=E _i_ [(T _i_/x _i_)-Z]. Z is the threshold budget share. The minimum and maximum value of catastrophic payment gap is 0% and 90% respectively when the threshold value is fixed at 0.10.

### Measuring socioeconomic inequalities of CHE

The measures of CHE are insensitive to household economic conditions and thus do not identify whether the poor or rich households exceed the threshold more ( O’Donnell *et al*., 2008). Many policymakers will consider it a significant problem if the poorer households exceed the threshold level compared to the richer households. Wagstaff *et al*. recommend the calculation of concentration indices to separate the association of CHE with socio-economic status ( Wagstaff *et al.,* 1989). Concentration indices are used to detect the presence of socioeconomic inequality in any health sector variable and whether it is more marked in one group than another ( Kakwani 1977; Kakwani *et al.*, 1997; Takayama and Nanack, 1983; Wagstaff *et al*., 1989).

### Prediction model of CHE

To investigate the effects of different factors on the incidence of CHE, the logistic regression model is used. A dichotomous variable for CHE is created with 0 for not incurring catastrophic health expenditures and 1 for incurring catastrophic health expenditures. Thus, the dichotomous variable created for CHE will serve as the dependent variable for the logistic regression model. Among the households which incurred CHE, intensity of CHE was calculated, and multiple regression model was used to identify factors affecting intensity levels. The dependent variable is the catastrophic payment gap, and the independent variables included various characteristics of the individuals, households and health facility. Globally many studies have those specific household characteristics such as location, size of the household, utilization of private health facilities, health insurance coverage, presence of chronic illnesses, hospitalizations, presence of elderly and children in the household ( Bhojani *et al*., 2012; Brinda *et al*., 2014; Dwivedi & Pradhan 2017; Mondal *et al*., 2014; Srivastava *et al*., 2009; Leive & Xu., 2008).

## Results

Descriptive statistics presented in Table 1. There were 65,932 households in the sample in the 2014 data and 113,823 households in the 2018 data. 33% of the households (2014) and 25% of households (2018) have at least one child aged 5 years and less; 26.87% households (2014) and 22.92% households (2018) have at least one elderly person. The proportion of households located in rural and urban areas are almost same in 2014 and 2018. About 9.98% of the households (2014) and 15.40% of households (2018) have members who used a private hospital.

**Table 1.** Descriptive statistics of the household characters.

Variables	Definition and categories	Weighted percentage - 2014	Weighted percentage - 2018
Age groups (Children)	Presence of at least one child (aged 5 years and less) in the household	33%	24.94%
Age groups (Elderly)	Presence of at least one elderly person (aged 60 years and above in the household	26.87%	22.92%
Marital status	Presence of someone divorced in the household	22.44%	20.98%
Female education	Presence of at least one secondary educated female member in the household	33.94%	41.01%
Location of the household	Rural	67.44%	67.24%
	Urban	32.56%	32.76%
Socioeconomic status of household	Lowest Income Quintile	30.04%	30.39%
	Second Lowest Income Quintile	21.77%	23.97%
	Third Income Quintile	20.59%	17.9%
	Fourth Income Quintile	15.59%	15.49%
	Highest Fifth Income Quintile	12.01%	12.24%
Household size	Small household (1 to 4 members)	54.08%	57.68%
	Medium household (5 to 8 members)	40.94%	38.56%
	Large household (9 and more)	4.98%	3.76%
Religion of the household	Hinduism	82.35%	82.60%
	Islam	12.59%	12.50%
	Christianity	2.34%	2.46%
	Other religions	2.72%	2.45%
Social group of the household	Scheduled tribes	9.14%	9.03%
	Scheduled castes	18.69%	19.22%
	Other backward classes	43.26%	44.51%
	Others	28.91%	27.24%
Level of care – among hospitalized	If at least one member in the household used a private healthcare facility for hospitalization	9.98%	15.40%

Variables	Definition	Mean - 2014 (Standard deviation)	Mean - 2018 (Standard deviation)
Sex	Proportion of female members in each household	0.48 (0.0018)	0.48 (0.0016)
Health Insurance coverage	Proportion of members enrolled in health insurance in each household	0.16 (0.0032)	0.17 (0.0027)
Chronic illness	Proportion of members suffering from chronic illness in each household	0.06 (0.0014)	0.04 (0.0009)
Hospitalization	Proportion of members hospitalized in each household	0.04 (0.0006)	0.03 (0.0004)
Duration of hospitalization	Total duration of hospitalization of all members in each household	1.29 days (0.025)	0.91 days (0.0141)
Monthly consumption expenditure	Total consumption expenditure of all members in each household per month	7333.01 INR (45.04)	9404.56 INR (44.68)
Total monthly OOP health expenditure	Total OOP health expenditures of all members in each household per month	403.43 INR (14.48)	681.01 INR (14.21)

* Sample size N = 65,932 (2014) and N= 113,823 (2018).

### Incidence of catastrophic health expenditures

Table 2 shows that the CHE incidence was 10.94% in 2014 and 16.51% in 2018. Incidence of CHE was 64.57% (2014) and 43.99% (2018) among households that used a private hospital. Table 3 shows that the mean positive overshoot indicates that on average, the OOP was 35.94% (2014) and 34.08% (2018).

**Table 2.** Incidence of CHE by household characteristics.

Variables	Categories	Incidence of CHE at 10% threshold level – 2014	Incidence of CHE at 10% threshold level – 2018	Percentage change in incidence of CHE – 2014 to 2018
Percent of total households reporting catastrophic health expenditures		10.94%	16.51%	+5.57%
Sector	Rural	11.17%	16.89%	+5.72%
	Urban	10.45%	15.72%	+5.27%
Household size	Small household (1 to 4 members)	9.14%	15.39%	+6.25%
	Medium household (5 to 8 members)	12.68%	17.72%	+5.04%
	Large household (9 and more)	16.15%	21.23%	+5.08%
Religion of the household	Hinduism	10.67%	16.02%	+5.35%
	Islam	12.36%	18.93%	+6.57%
	Christianity	12.22%	16.72%	+4.50%
	Other religions	11.49%	20.52%	+9.03%
Social group of the household	Scheduled tribes	7.13%	12.18%	+5.05%
	Scheduled castes	10.52%	15.84%	+5.32%
	Other backward classes	11.28%	16.20%	+4.92%
Private healthcare facility for hospitalization	If at least one member in the household used a private healthcare facility	64.57%	43.99%	-20.58%
	No member in the household used a private healthcare facility	4.99%	11.51%	+6.52%
Child aged 5 years and less in the household	At least one child aged less than 5 years present in the household	14.49%	19.86%	+5.37%
	No child less than 5 years in the household	9.19%	15.40%	+6.21%
Elderly aged 60 years and above	At least one elderly person aged 60 years and above in the household	15.43%	27.30%	+11.87%
	No elderly aged 60 years and above in the household	9.29%	13.30%	+4.01%
Secondary educated female in household	At least one secondary educated female member in the household	12.71%	18.17%	+5.46%
	No secondary educated female member in the household	10.03%	15.36%	+5.33%
Divorced person in household	At least one divorced person in the household	12.72%	20.88%	+8.16%
	No divorced person in the household	10.42%	15.35%	+4.93%

CHE: Catastrophic Health Expenditure.

**Table 3.** Intensity of CHE by household characteristics.

Variables	Categories	CHE overshoot – 2014	CHE overshoot – 2018	Percentage change – 2014 to 2018
Mean positive overshoot		35.94%	34.08%	-1.86%
Sector	Rural	36.91%	35.82%	-1.09%
	Urban	33.78%	30.25%	-3.53%
Household size	Small household (1 to 4 members)	42.76%	38.99%	-3.77%
	Medium household (5 to 8 members)	31.18%	28.34%	-2.84%
	Large household (9 and more)	24.74%	28.59%	+3.85%
Religion of the household	Hinduism	35.81%	34.48%	-1.33%
	Islam	34.81%	30.97%	-3.84%
	Christianity	45.44%	36.48%	-8.96%
	Other religions	36.50%	36.38%	-0.12%
Social group of the household	Scheduled tribes	63.99%	33.61%	-30.38%
	Scheduled castes	36.03%	38.98%	+2.95%
	Other backward classes	32.96%	34.13%	+1.17%
	Others	34.80%	31.21%	-3.59%
Private healthcare facility for hospitalization	If at least one member in the household used a private healthcare facility	34.07%	37.26%	+3.19%
	No member in the household used a private healthcare facility	38.62%	31.87%	-6.75%
Child aged 5 years and less in the household	At least one child aged less than 5 years present in the household	26.27%	26.27%	0%
	No child less than 5 years in the household	43.45%	37.43%	-6.02%
Elderly aged 60 years and above	At least one elderly person aged 60 years and above in the household	41.28%	37.10%	-4.18%
	No elderly aged 60 years and above in the household	32.68%	32.24%	-0.44%
Secondary educated female in household	At least one secondary educated female member in the household	30.50%	33.77%	+3.27%
	No secondary educated female member in the household	39.48%	34.34%	-5.14%
Divorced person in household	At least one divorced person in the household	43.45%	38.40%	-5.05%
	No divorced person in the household	33.29%	35.96%	+2.67%

CHE: Catastrophic Health Expenditure.

### Incidence and intensity of catastrophic health expenditures by state

**Figure 2.**
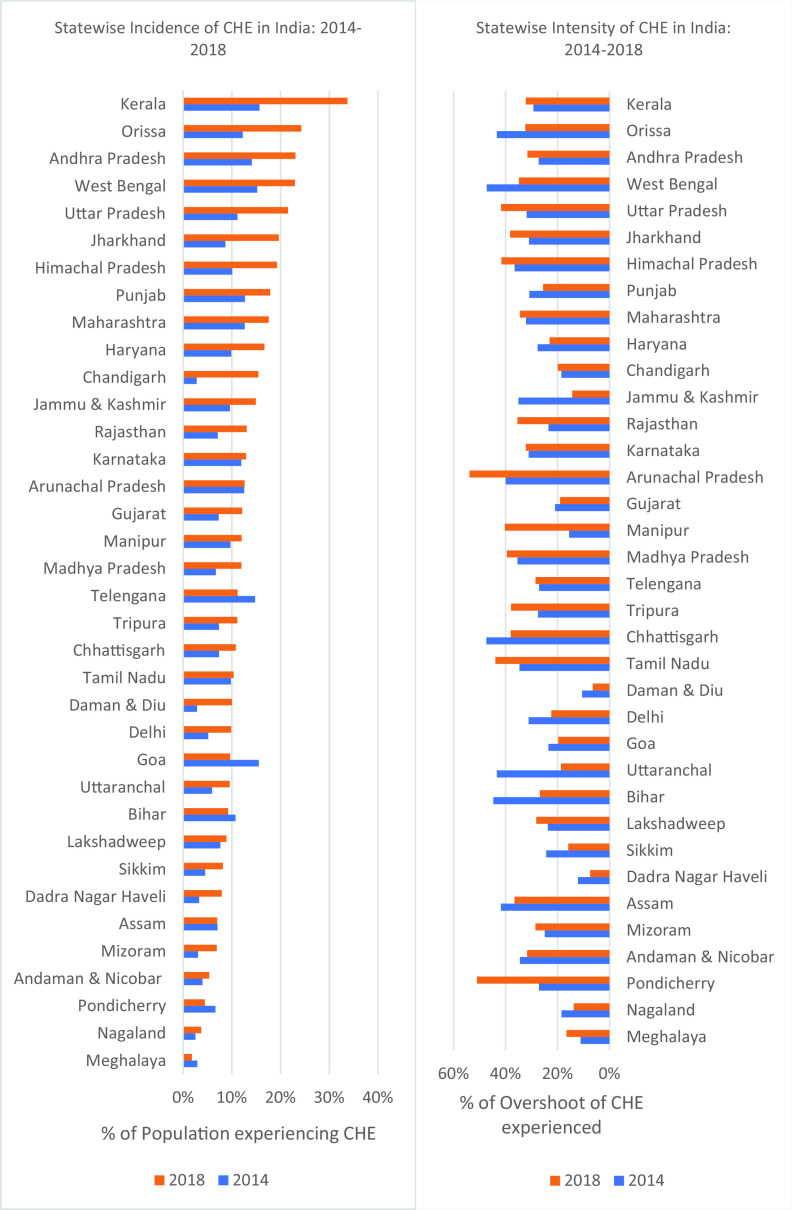
Incidence and intensity of CHE by state.

**Figure 3.**
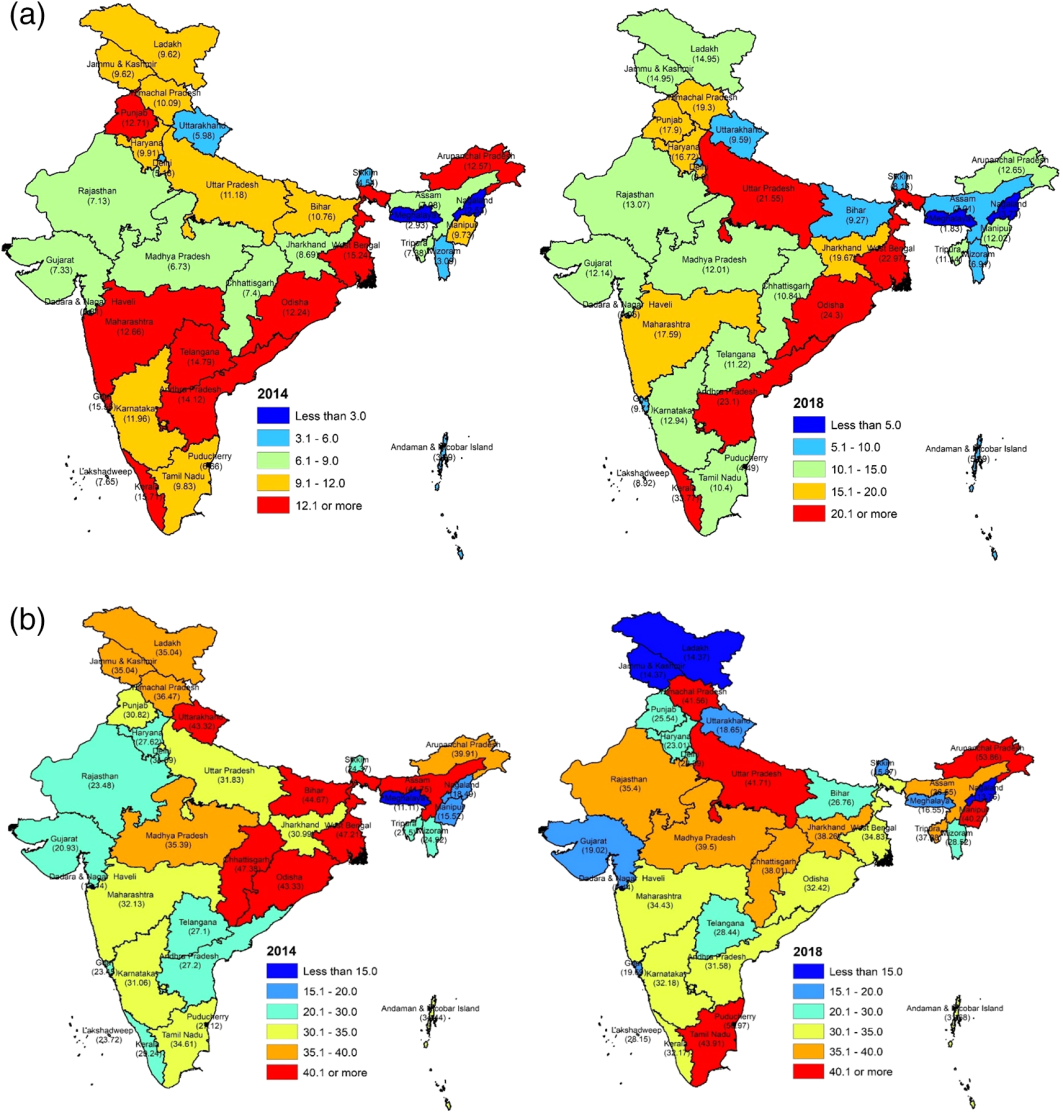
a: Map showing incidence of CHE by states, 2014 and 2018. b: Map showing intensity of CHE by states, 2014 and 2018.

**Figure 4.**
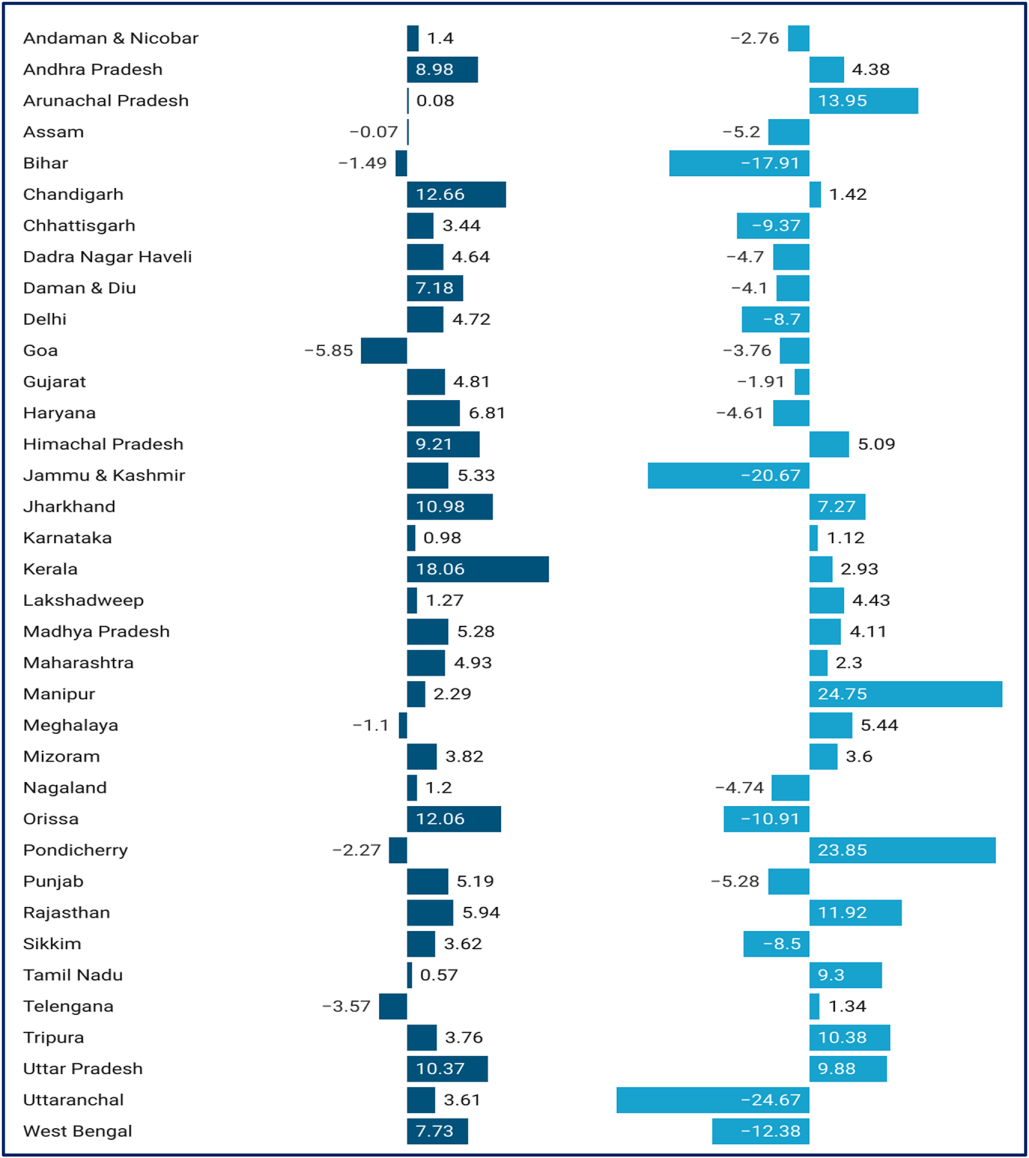
Change in incidence and intensity of CHE by states: 2014-2018.

Figure 2 and Figure 3a show the incidence of CHE for the states in India for the years 2014 and 2018. Figure 2 and Figure 3b show the intensity of CHE for the states in India for the years 2014 and 2018. Figure 4 shows the change in CHE between 2014 to 2018 across the different states. In 2018, Kerala (33.77%) has the highest incidence of CHE, while Meghalaya (1.83%) has the lowest incidence of CHE. In 2014, Kerala (15.71%) has the highest incidence of CHE, while Chandigarh (2.80%) has the lowest incidence of CHE. In 2018, Arunachal Pradesh (53.86%) has the highest intensity of CHE, while Daman and Diu (6.45%) has the lowest intensity of CHE. In 2014, Chhattisgarh (47.38%) has the highest intensity of CHE, while Daman and Diu (10.55%) has the lowest intensity of CHE. Kerala (18.06%) experienced the highest increase in incidence of CHE from 2014 to 2018, while Goa (5.85%) experienced a decline in incidence of CHE from 2014 to 2018. Manipur (24.75%) experienced the highest increase in intensity of CHE from 2014 to 2018, while Uttaranchal (24.67%) experienced a decline in intensity of CHE from 2014 to 2018. In 2014, households in the richest expenditure quintile have the highest incidence of CHE, while in 2018, households all the income expenditure quintiles incur almost more or less same incidence of CHE as shown in Table 4.

**Table 4.** Headcount and overshoot of CHE by socioeconomic status and location.

Threshold	2014			2018		
	Rural	Urban	Total	Rural	Urban	Total
**Headcount**						
Poorest	9.61%	7.33%	9.22%	15.98%	13.86%	15.64%
Second	10.51%	8.68%	10.05%	17.28%	16.52%	17.12%
Third	10.93%	10.24%	10.71%	16.54%	17.75%	16.94%
Fourth	14.24%	12.66%	13.50%	18.09%	16.24%	17.18%
Richest	18.61%	11.70%	13.82%	20.70%	14.57%	16.00%
CI	0.0910	0.0904	0.0848	0.0268	-0.0078	0.0113
SE (CI)	0.0081	0.0096	0.0062	0.0048	0.0058	0.0037
**Overshoot**						
Poorest	60.20%	43.92%	58.03%	47.68%	54.70%	48.67%
Second	31.12%	43.78%	33.84%	31.20%	39.04%	32.80%
Third	23.75%	28.21%	25.10%	30.46%	26.30%	29.04%
Fourth	21.76%	28.81%	24.86%	25.09%	24.24%	24.70%
Richest	28.37%	32.92%	31.04%	23.36%	21.27%	21.90%
MPO	36.91%	33.78%	35.94%	35.82%	30.25%	34.08%
CI	-0.1328	0.0277	-0.0882	-0.1089	-0.1836	-0.1440
SE (CI)	0.0242	0.0239	0.0177	0.0128	0.0220	0.0111

CHE: Catastrophic Health Expenditure; CI: Concentration Index; SE: Standard Error; MPO: Mean Positive Overshoot.

### Multivariate analysis

Table 5 shows that both in 2014 and 2018, it was observed that the odds of experiencing CHE was higher among households with children, elderly person, and utilization of a private hospital. Urban households had lower probability of experiencing incidence of CHE, and households from all other expenditure quintiles also had lesser odds of incurring CHE compared to households in the poorest quintile both in 2014 and 2018. The likelihood of incidence of CHE increased with the increase in duration of stay in the hospital, with the highest odds being for the households who had members who stayed for more than 20 days in a hospital. Also, the presence of chronic illness among members in the household increased odds of CHE. Health insurance coverage in the household reduced the likelihood of CHE incidence. Table 6 shows that both in 2014 and 2018, households’ children aged less than 5 years, members being covered by health insurance, and not belonging to the poorest expenditure quintile had lower intensity of CHE.

**Table 5.** Logistic regression for the factors affecting incidence of CHE.

Characteristics	2014 (n = 65,932)		2018 (n = 113,823)	
	Odds ratio (95% CI)	P value	Odds ratio (95% CI)	P value
At least one member in the household has health insurance coverage	0.62	0.000	0.82	0.001
	(0.52-0.75)		(0.73-0.92)	
Presence of at least one elderly aged more than 60 years present in the household	1.27	0.002	1.47	0.000
	(1.09-1.48)		(1.33-1.62)	
Presence of someone divorced in the household	0.94	0.467	1.01	0.950
	(0.82-1.09)		(0.90-1.11)	
Presence of at least one child aged less than 5 years in the household	1.34	0.000	1.02	0.562
	(1.18-1.52)		(0.93-1.12)	
**Sector**				
Rural (Reference)				
Urban	0.91	0.192	0.91	0.043
	(0.81-1.04)		(0.83-0.99)	
**Socioeconomic status**				
Poorest Expenditure Quintile (Reference)				
Second Lowest Expenditure Quintile	0.74	0.001	0.99	0.953
	(0.62-0.88)		(0.88-1.11)	
Third Expenditure Quintile	0.60	0.000	0.81	0.002
	(0.50-0.73)		(0.72-0.92)	
Fourth Expenditure Quintile	0.51	0.000	0.68	0.000
	(0.41-0.65)		(0.60-0.79)	
Highest Fifth Expenditure Quintile	0.28	0.000	0.47	0.000
	(0.21-0.38)		(0.40-0.55)	
**Household size**				
Small household (Reference)				
Medium household (5 to 8)	0.95	0.543	1.28	0.000
	(0.83-1.10)		(1.17-1.40)	
Large household (9 & more)	0.68	0.011	1.25	0.013
	(0.50-0.91)		(1.17-1.40)	
**Duration of hospitalization**				
Less than 5 days (Reference)				
5 to 10 days	8.41	0.000	4.05	0.000
	(7.46-9.49)		(3.73-4.39)	
11 to 20 days	16.42	0.000	7.19	0.000
	(13.64-19.76)		(6.34-8.15)	
More than 20 days	48.92	0.000	8.62	0.000
	(37.93-63.10)		(6.11-12.14)	
At least one member in the household used a private healthcare facility	28.21	0.000	4.16	0.000
	(24.57-32.38)		(3.85-4.49)	
At least one person in the household suffers from chronic illness	3.11	0.000	4.15	0.000
	(2.65-3.64)		(3.55-4.84)	
Constant	0.07	0.000	0.09	0.000
	(0.05-0.12)		(0.07-0.10)	

**Table 6.** Multiple regression for the factors affecting intensity of CHE.

Characteristics	2014 (n = 65,932)		2018 (n = 113,823)	
	Coef. (95% CI)	P value	Coef. (95% CI)	P value
Proportion of members having health insurance coverage in each household	-1.88	0.013	-7.16	0.021
	(-3.36 - -0.40)		(-13.24 - -1.08)	
Presence of at least one child aged less than 5 years present in the household	-7.06	0.001	-6.24	0.000
	(-11.11 - -3.01)		(-9.72 - -2.75)	
Presence of at least one elderly aged more than 60 years present in the household	3.74	0.402	3.11	0.207
	(-5.02-12.52)		(-1.72 - 7.96)	
Presence of someone divorced in the household	8.83	0.142	-3.73	0.088
	(-2.96-20.63)		(-8.03 - 0.55)	
**Sector**				
Rural (Reference)				
Urban	1.11	0.668	-0.49	0.818
	(-3.96-6.18)		(-4.72 - 3.73)	
**Socioeconomic status**				
Poorest Expenditure Quintile (Reference)				
Second Lowest Expenditure Quintile	-20.58	0.000	-16.32	0.000
	(-31.15 - -10.01)		(-23.29 - -9.34)	
Third Expenditure Quintile	-28.77 (-37.15 - -20.38)	0.000	-22.00 (-29.53 - -14.47)	0.000
Fourth Expenditure Quintile	-30.46	0.000	-28.30	0.000
	(-38.92 - -22.00)		(-36.31 - -20.29)	
Highest Fifth Expenditure Quintile	-27.78	0.000	-35.37	0.000
	(-37.23 - -18.34)		(-43.63 - -27.12)	
**Household size**				
Small household (Reference)				
Medium household (5 to 8)	-3.02	0.412	-0.99	0.748
	(-10.24-4.19)		(-7.03 - 5.05)	
Large household (9 & more)	-7.55	0.116	3.55	0.360
	(-16.95-1.85)		(-4.05 - 11.16)	
**Duration of hospitalization**				
Less than 5 days (Reference)				
5 to 10 days	6.54	0.000	18.14	0.000
	(3.38-9.71)		(12.81 - 23.46)	
11 to 20 days	25.61	0.000	33.42	0.000
	(20.96-30.25)		(28.50 - 38.33)	
More than 20 days	64.66	0.000	72.06	0.000
	(51.30-78.03)		(58.45 - 85.68)	
Proportion of members with chronic illness in each household	34.29	0.006	26.37	0.000
	(10.06-58.52)		(14.05 - 38.69)	
Constant	46.50	0.001	41.91	0.000
	(19.30-73.69)		(32.38 - 51.45)	

## Discussion

In 2014, among the households which experienced CHE, the mean positive overshoot indicates that on average, the OOP health expenditures was 35.94% higher, but the overshoot decreased to 34.08% in 2018. This shows that although the intensity is very high among the households experiencing CHE, but it is decreasing. Our study showed that both in 2014 and 2018, a higher odds of incidence of CHE among the households with children, elderly people and those who used a private hospital for treatment. This was consistent with literature which showed that households which consisted of members at extremes of age ( Mohanty *et al*., 2014; da Silva *et al*., 2015), members utilization of private health facility ( Alam & Mahal 2014; Saksena *et al*., 2012; Somkotra & Lagrada, 2009) had higher OOP and CHE. The likelihood of incidence and intensity of CHE in our study increased progressively with the increase in duration of hospital stay. However, the effect of duration of hospitalization on incidence of CHE was much lower in 2018 compared to 2014, but the intensity of CHE was much higher in 2018 compared to 2014. This shows that health insurance and other health financing protection mechanisms may have been successful in reducing the number of households being pushed to experience CHE, but among the households that experienced CHE, the burden (overshoot) has increased greatly over this time-period. A World Bank study showed that hospitalizations are the major drivers of OOP health expenditures ( McIntyre *et al*., 2006). Also, the presence of chronic illness among members in the household increased odds of CHE incidence and also increased the intensity among households experiencing CHE. Similar results were found in India ( Mondal *et al*., 2014), Bangladesh ( Molla *et al*., 2017), and China ( You & Kobayashi, 2011) that showed that chronic illness is an important determinant for experiencing CHE. Our study showed that the presence of health insurance coverage among members in the household reduced the likelihood of CHE incidence and even among the households that experienced CHE, the intensity was lesser for households that had health insurance coverage. Other studies from India ( Fan *et al*., 2012), Indonesia ( Aji *et al*., 2013), Laos ( Alkenbrack & Lindelow; 2015), and Vietnam ( Sepehri 2013) supported this finding of the protective effect of health insurance from CHE.

The regression results show that the households from all other expenditure quintiles had lesser odds of incurring CHE compared to poorest households. Among the households that experienced CHE, the intensity was also highest among the poorest households. For the poorer households, high level of intensity or overshoot may be due to low level of absolute income. It is expected that the poor people are more prone to experience CHE, since they have lower level of income and any expenditure that incur for healthcare will easily make it “catastrophic” since the proportion of the health expenditure will become relatively high for them because of low total consumption expenditure (low value of denominator). Thus, poorest people have higher risk of facing CHE even with a relatively small adverse health event. Globally there is mixed evidence on the relationship between socio-economic status and CHE. These results are similar with the findings from research done in India ( Bhojani *et al*., 2012), Thailand, Paraguay, and Burkina Faso ( Makinen *et al*., 2000) which showed that low-income households were associated with a higher likelihood of CHE. Other studies in Nigeria, Namibia, Albania, Kenya, Bangladesh, and India show that poorer households have lower absolute OOP health expenditures compared to richer individuals and households, but the relative proportion of OOP health expenditures to non-food household expenditures was higher in poor households ( Chuma & Maina 2012; Gustafsson-Wright *et al*., 2011; Hotchkiss *et al*., 2005; Karan *et al*., 2014; Onwujekwe *et al*., 2014; Rahman *et al*., 2013). However, studies from 13 low-income Asian countries ( O'Donnell O *et al*. 2008), Sri Lanka, South Africa and Guatemala ( Makinen *et al*., 2000) showed that richer households spent more on OOP health expenditures and also enjoyed a wide range of services. There is an increase in both the incidence and intensity of CHE with higher duration of hospital stay. Higher duration of hospital stay increases the chance of experiencing CHE. When the higher health expenditures are inadequately covered by health insurance, OOP health expenditures may become catastrophic for many households.

There are wide differences among states of India in CHE experienced by them. Epidemiological transition and economic growth also lead to an increase in CHE. Higher economic growth leads to an increase in demand for healthcare services and increase in technology used in healthcare delivery. For example, Kerala being an economically advanced state experienced higher incidence of CHE. States like Meghalaya and Nagaland which are economically poor experienced lower incidence of CHE. In the developed states of India, the higher CHE may be due to increased awareness of health benefits and increased utilization of private healthcare facilities over the free public health facilities. The epidemiological and disease burden in a state also determines the healthcare utilization, type of healthcare used, and the volume of healthcare services used in the state. For example, some economically better states may suffer from higher burden of non-communicable diseases, while in contrary some of the poorer states may suffer from maternal and child health problems and infectious diseases. The type of disease experienced by the states directly determines the health expenditures experienced by them ( Wagstaff *et al*., 2018; Kea *et al*., 2011; Kien *et al*., 2016).

Also, in India there is a very poor non-communicable disease service provision in the public sector, and this makes more people choose the private sector hospitals for non-communicable disease treatment which increases their OOP costs when not adequately covered by any health insurance. Different state health insurance programs increased the usage of more private healthcare facilities by increasing the access to them, they have not actually protected the households from the financial burden that arises due to the usage of private health facilities ( Karan *et al*., 2017; Selvaraj & Karan, 2012; Ranjan *et al*., 2018).

In conclusion, this research helps policymakers to design health insurance programs to better serve the population. Health insurance benefit packages and coverage limits may be adjusted based on the income levels of poor households with the poorest group receiving the highest level of protection. This type of targeting is also difficult to implement in practice, but it is not impossible with help from community organizations representing the poor and extremely poor households. Households with children less than 5 years and elderly more than 60 years have higher CHE incidence. Children and elderly are the vulnerable age groups who are prone to higher level of health risks. They have higher healthcare utilizations and thus experience higher healthcare expenditures which make the expenditure levels catastrophic in many cases. This implies that policymakers should also consider age as an important factor for health insurance coverage. Health insurance coverage limits in India are restricted and are not adequate especially when the patients stay for longer duration in the hospitals. Thus, the coverage limits for hospital insurance needs to be increased to protect households from CHE. Also, better cost regulation of the private sector hospitals must be done. Chronic illness increases both CHE incidence and intensity. Steps should be taken for early diagnosis and treatment, to reduce the severity of illness, reduce the cost of services, and implementation of better approaches to treat them in the ambulatory settings. This research will help in developing policies to reduce OOP health expenditures in India.

## Author contributions

SS was involved in Conceptualization, Data Curation, Formal Analysis, Investigation, Methodology, Project Administration, Resources, Software, Supervision, Validation, Visualization, Writing – Original Draft Preparation, Writing – Review & Editing. MA was involved in Writing – Original Draft Preparation, Writing – Review & Editing.

## Data availability

The data from the National Sample Survey Organization (NSSO) of the Government of India were used for the study. Social Consumption (Health), NSS 71 ^st^ Round for 2014 and NSS 75 ^th^ Round for 2018 were used for this analysis. Both the surveys covered whole of the Indian Union. Data can be obtained from the Government of India or from the corresponding author by reasonable request.
