# Supplementary material for: A STUDY OF CATASTROPHIC HEALTH EXPENDITURES IN INDIA - EVIDENCE FROM NATIONALLY REPRESENTATIVE SURVEY DATA: 2014-2018
Source: F1000Res. 2022 Feb 3;11:141. [Version 1] doi: 10.12688/f1000research.75808.1 (PMC9005991; doi:10.12688/f1000research.75808.1)
Supplement: Peer review history

# Peer review: Reviewer response for version 1

**DOI:** 10.5256/f1000research.79730.r122490

**Published:** 2022-04-12

**Authors:** Shabana Tharkar

**Affiliations:** 1 Prince Sattam Chair for Epidemiology and Public Health Research, College of Medicine, King Saud University, Riyadh, Saudi Arabia

The manuscript is well written. The author must be applauded for their magnanimous efforts. The study provides useful information to the public health officials and lawmakers in formulating or revising policies and programs incorporating those identified at high risk. The study identifies the determinants of catastrophic health expenditure suggesting the type of population that is most susceptible. The insurance schemes whether public or private can apply these findings to include the vulnerable households in their coverage. Dissemination of the findings may prove valuable to the Health Ministry in initiating relevant programs for the betterment of the vulnerable.

 Minor change required:

 The first line of the fourth paragraph, under discussion section - *“Also, in India there is a very poor non-communicable disease service provision in the public sector, and this makes more people choose the private sector hospitals for non-communicable disease treatment which increases their OOP costs when not adequately covered by any health insurance.”* must be reframed in a more polite language and supported by evidence.

 Otherwise the manuscript can be indexed as such.

 Thank you.

Is the work clearly and accurately presented and does it cite the current literature?

Yes

If applicable, is the statistical analysis and its interpretation appropriate?

Yes

Are all the source data underlying the results available to ensure full reproducibility?

Yes

Is the study design appropriate and is the work technically sound?

Yes

Are the conclusions drawn adequately supported by the results?

Yes

Are sufficient details of methods and analysis provided to allow replication by others?

Yes

Reviewer Expertise:

Non communicable disease epidemiology

I confirm that I have read this submission and believe that I have an appropriate level of expertise to confirm that it is of an acceptable scientific standard.

# Peer review: Reviewer response for version 1

**DOI:** 10.5256/f1000research.79730.r122493

**Published:** 2022-02-17

**Authors:** Harsh D. Shah

**Affiliations:** 1 Indian Institute of Public Health Gandhinagar (IIPHG), Gandhinagar, Gujarat, India

The countries should design or customize their health insurance schemes based on pieces of evidence generated through country-specific research studies. The present article has provided key focus areas with stratification of incidence and intensity of catastrophic health expenditure (CHE) of the specific sections of the population and identified the areas where public insurance schemes can concentrate their expansion of existing interventions. 

 The article fulfills the criteria for bringing new information, built on replicable methodology, covers the results as intended in objectives and fits in the criteria for research indexing approval. 

**Is the work clearly and accurately presented and does it cite the current literature?** - Partly. 

 The article has supportive references to direct the study findings, but I cannot find references published after the year 2019. The literature search options should be expanded with recent years. The present study does specify the incidence and intensity of CHE with comparison among different strata with statistical analysis through a regression model. The other pieces of literature have not reflected on the same objective and this way, the article shows its uniqueness. 

**Abstract Section of Conclusion:** *"... conduct regular audits to ensure that private hospitals do not increase hospitalizations and the duration of stay is necessary."* : Avoid the statement without provision of references.

**Are the conclusions drawn adequately supported by the results?** Partly.

*"Also, in India there is a very poor non-communicable disease service provision in the public sector, and this makes more people choose the private sector hospitals for non-communicable disease treatment which increases their OOP costs when not adequately covered by any health insurance"* - Reference is required if it is not a study finding.

Is the work clearly and accurately presented and does it cite the current literature?

Partly

If applicable, is the statistical analysis and its interpretation appropriate?

Yes

Are all the source data underlying the results available to ensure full reproducibility?

Yes

Is the study design appropriate and is the work technically sound?

Yes

Are the conclusions drawn adequately supported by the results?

Partly

Are sufficient details of methods and analysis provided to allow replication by others?

Yes

Reviewer Expertise:

Public Health Research, Operational Research, Diseases Outbreaks, Maternal and Child Health, Epidemiology, Program Evaluation, Health Policy and Financing, Health Economics,

I confirm that I have read this submission and believe that I have an appropriate level of expertise to confirm that it is of an acceptable scientific standard.

## References

[1] : Effect of health insurance program for the poor on out-of-pocket inpatient care cost in India: evidence from a nationally representative cross-sectional survey. *BMC Health Serv Res* .2020;20(1) : 10.1186/s12913-020-05692-7 839 10.1186/s12913-020-05692-7 32894118PMC7487854

[2] : Role of insurance in determining utilization of healthcare and financial risk protection in India. *PLoS One* .2019;14(2) : 10.1371/journal.pone.0211793 e0211793 10.1371/journal.pone.0211793 30721253PMC6363222
